# Stability and Compatibility Aspects of Drugs: The Case of Selected Cephalosporins

**DOI:** 10.3390/antibiotics10050549

**Published:** 2021-05-09

**Authors:** Szymon Tomczak, Aleksandra Gostyńska, Malwina Nadolna, Karolina Reisner, Marta Orlando, Anna Jelińska, Maciej Stawny

**Affiliations:** 1 Department and Chair of Pharmaceutical Chemistry, Poznan University of Medical Sciences, 6 Grunwaldzka, 60-780 Poznań, Poland; szymon.tomczak@ump.edu.pl (S.T.); agostynska@ump.edu.pl (A.G.); mnadolna@wp.pl (M.N.); kwelnic@wp.pl (K.R.); ajelinsk@ump.edu.pl (A.J.); 2 Department of Chemical, Biological, Pharmaceutical and Environmental Sciences, University of Messina, Viale F. Stagno D’Alcontres 31, I-98166 Messina, Italy; orlando.marta@hotmail.it

**Keywords:** compatibility, stability, cephalosporins, parenteral nutrition

## Abstract

Intravenous drug incompatibilities are a common cause of medical errors, contributing to ineffective therapy and even life-threatening events. The co-administration of drugs must always be supported by studies confirming compatibility and thus guarantee the therapy’s safety. Particular attention should be paid to the possible incompatibilities or degradation of intravenous cephalosporins in different infusion regimens since the administration of drugs with inadequate quality may cause treatment failure. Therefore, an appropriate stability test should be performed. The study aimed to present various aspects of the stability and compatibility of five cephalosporins: cefepime (CFE), cefuroxime (CFU), ceftriaxone (CFX), ceftazidime (CFZ), and cefazoline (CFL). The degradation studies in parenteral infusion fluids and PN admixtures were conducted for CFE and CFU. The interactions between CFX or CFZ and PN admixtures, as well as the compatibility of CFL with five commercial parenteral nutrition (PN) admixtures, were investigated. The content of CFX and CFZ in PN admixture after 24 h was >90%. CFL administered simultaneously with PN admixture by the same infusion set using Y-site was compatible only with Nutriflex Lipid Special. CFE and CFU were stable in all tested infusion fluids for a minimum of 48 h and decomposed in PN admixtures during storage.

## 1. Introduction

Injectable drugs must meet special requirements. Apart from sterility and apyrogenicity, they must be free from solid contamination [1]. Drugs in the form of emulsions for injection must correspond to pharmacopoeial requirements for the particle size of the lipid droplets. The intensity-weighed mean droplet diameter (MDD) of lipid emulsion must be less than 500 nm, and the volume-weighted percentage of lipid droplets above 5 µm (PFAT5) cannot exceed 0.05% [2]. The simultaneous administration of two or more drugs may cause incompatibility between them, disqualifying such therapies. Although a limited number of medications are administered intravenously, such drugs account for the majority of medication errors contributing to ineffective therapy and even life-threatening events [3,4,5]. The most common signs of incompatibilities are precipitation, turbidity, discoloration, or destabilization of dispersed systems, which can be a consequence of the influence on the stability of the drug substance such as oxidation, racemate formation, hydrolysis, or condensation [6]. The co-administration of two or more drugs using the same infusion line or mixed together in one container must always be supported by studies that confirm the compatibility of such infusion regimens and thus guarantee the safety of the therapy [6,7,8,9].

Compatibility of drugs and parenteral nutrition (PN) admixtures is an exceptional case of intravenous drug interactions [10,11]. Precipitation or incompatibility, affecting the particle size of the emulsion, invisible to the human eye, can have fatal consequences [12,13,14]. The PN admixture should be considered as a drug consisting of even several dozen ingredients (water, glucose, electrolytes, vitamins, trace elements, emulsifiers, lipids) mixed and stored in one container [15]. The PN admixture can cause many drug incompatibilities due to the content of calcium and magnesium ions, thereby precipitating insoluble salts [14,15,16,17], as well as accelerating the degradation of the drug by the presence of trace elements that can catalyze decomposition reactions [18,19].

Interaction studies between drugs and reconstitution fluids or PN admixture can be studied in two different protocols. The first one is the addition of the reconstituted drug to infusion fluids to store for a longer time [20,21,22,23,24,25,26] or, in the case of PN admixture, the use of such medium as a drug carrier [27,28,29,30,31,32]. The second study protocol assumes administering two infusion drugs through the same infusion set using a Y-site connector [11,33,34,35,36,37]. In this case, the contact time between drugs is very short and limited only to the common part of the infusion line. These differences affect the research methodology. In the first case, it is required to confirm both the compatibility, understood as no turbidity or precipitate, and stability understood as maintaining drug content on the level of more than 90% of nominal content [22,38,39,40]. Long-term stability studies are conducted in different conditions of temperature and light access. Due to the long administration time of PN admixtures, it is required that the stability of added drug will be maintained at least 24 h to ensure a proper dose of the drug and not to expose the patient to the drug degradation products. In the second case, stability studies are not necessary [41,42,43]. Here, attention should be paid to the compatibility of such a combination. Possible interactions between drugs may occur only in a common part of the infusion line just before administration to the patient’s bloodstream. Hence, the time for the possible chemical reaction between both pharmaceutical preparation is very short, and thus such reactions are limited. Physicochemical incompatibilities of parenteral drugs and PN admixtures can be detected by visual inspection, and pH, osmolality, zeta potential, size of lipid emulsion particles measurements [44,45,46,47,48].

There is still no consensus in the literature about which tests should be performed to assess drug−drug incompatibilities, especially concerning infusion drugs and PN admixtures. Appropriate analytical methods and acceptance criteria allowing detection of drug−drug interactions are difficult to define. Methods applied in this study were chosen based on our own experience and literature data and should enable us to detect both signs of precipitation and emulsion destabilization [34]. The purpose of the study was to present various aspects of the stability and compatibility of five intravenous cephalosporins cefepime (CFE), cefuroxime (CFU), ceftriaxone (CFX), ceftazidime (CFZ), and cefazoline (CFL) with PN admixtures and/or parenteral infusion fluids. Three different drug−drug compatibility and stability aspects were undertaken. The first was the assessment of the stability of drugs (CFE, CFU, CFX, and CFZ) and compounded PN admixtures stored in one packaging in different storage conditions. The following analytical methods were applied to assess the compatibility and stability of cephalosporins in PN admixtures: pH-meter, visual inspection, zeta potential, osmolality, MDD, and HPLC. Second, stability studies in parenteral infusion fluids were performed for antibiotics that proved to be unstable in PN admixture (CFE and CFU). Third, the possibility of co-administration of CFL with commercial PN admixtures using Y-site was investigated. In this study, the extreme mixing ratios and resulting therefrom extreme concentration ranges, which were calculated based on the infusion rates during Y-site administration, were applied.

## 2. Results

### 2.1. Stability of CFE and CFU in Parenteral Infusion Fluids and PN Admixtures

The stability of CFE and CFU were determined in five parenteral infusion fluids: Water for injection, 5% glucose, 10% glucose, 0.9% sodium chlorate, Ringer’s solution, and in compounded PN admixture. CFE and CFU appeared as a white or slightly yellowish crystalline powder, freely soluble in water. The results from the UV spectra for water solution of both cephalosporins in the range from 220 to 400 nm indicated a clear maximum absorption of 272 nm. Therefore, this wavelength was used to monitor the changes in their concentration using the HPLC method. The obtained results indicate that CFE and CFU were the least stable in PN admixtures. The recovery of the drugs after 24 h of storage at the temperature of 5 ± 1 °C was 86.10% and 74.57% for CFE and CFU, respectively. For samples stored at the temperature of 25 ± 2 °C, the recovery was 73.71% and 73.87% for CFE and CFU, respectively. After five days of storage, the content of the drug decreased to 77.18% and 54.21% for CFE stored at the temperature of 5 ± 1 °C and 25 ± 2 °C, respectively. Results obtained in the same timeframes for CFU showed the reduction in the drug content to 38.27% and 24.65% for samples stored at the temperature of 5 ± 1 °C and 25 ± 2 °C, respectively. The highest drug recovery after 24 h of storage was obtained for CFE in Ringer’s solution (5 ± 1 °C) and in 10% glucose solution (25 ± 2 °C), while for CFU in 5% glucose solution (5 ± 1 °C) and water for injection (25 ± 2 °C). A time-dependent decrease in the content of both drugs was observed, as shown in Table 1.

### 2.2. The Interaction between CFX I CFZ and PN Admixtures

The aqueous solutions of CFX and CFZ were yellow. The PN admixture slightly turned yellow after the addition of the drug. No signs of emulsion decomposition were observed, while the color of the drug solutions stored at the temperature of 25 ± 2 °C gained a more intense yellow color, showing a clear difference to the samples stored at the temperature of 5 ± 1 °C.

**Figure 1:**
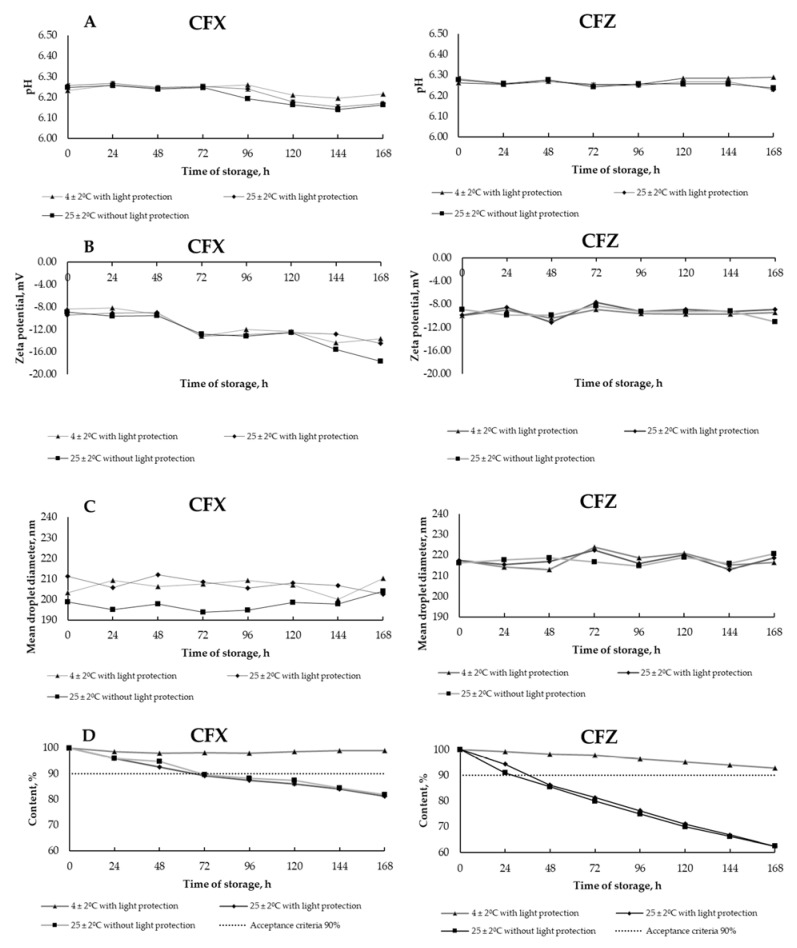
Results for ceftriaxone (CFX) and ceftazidime (CFZ) analysis: (**A**)—pH measurements, (**B**)—zeta potential, (**C**)—mean droplet diameter, and (**D**)—drugs degradation during storage in different conditions.

The pH of the PN admixtures immediately after preparation was 6.28 ± 0.01, and it changed slightly regardless of the storage conditions. The observed pH values during storage ranged from 6.27 ± 0.01 to 6.34 ± 0.01. The addition of CFX decreased the pH to the value of 6.23 ± 0.01, while CFZ did not affect this parameter. No significant changes in pH were observed at subsequent measuring points (Figure 1A). The zeta potential for PN admixtures immediately after preparation was −4.84 mV. The addition of CFX and CFZ reduced the zeta potential to −8.32 and −9.93 mV, respectively. A reduction in the zeta potential was observed at predetermined measurement points for both PN admixtures samples containing CFX and CFZ (Figure 1B). The MDD of the PN admixtures ranged from 200.7 ± 3.4 to 229.6 ± 10.3. The addition of the tested drugs did not cause any physicochemical changes manifested by the aggregation of the lipid emulsion particles. The MDD values for PN admixtures with CFZ ranged from 212.8 ± 7.9 to 224.0 ± 4.5, and for PN admixtures containing CFX ranged from 193.7 ± 4.5 to 213.7 ± 1.3. The MDD values for all tested samples throughout the study period were below 500 nm, and only one fraction of lipid particles was observed (Figure 1C).

A time-dependent degradation of CFZ and CFX in the PN admixture was observed. After 24 h, the loss of CFZ content was over 5% and 9%, for samples stored at the temperature of 25 ± 2 °C with and without light protection, respectively. For CFX, the content of the drug decreased below 10% in the first 24 h, regardless of storage conditions. The results of degradation of CFX and CFZ in PN admixtures at the assumed timeframe are shown in Figure 1D.

### 2.3. Compatibility of CFL and Ready-to-Use PN Admixture

The compatibility studies of CFL were conducted with five PN admixtures: Kabiven 1540 mL (Kabiven), Smofkabiven 1477 mL (Smofkabiven), Olimel N9E 1500 mL (Olimel), Nutriflex Omega Special 1875 mL (Nutriflex OS), Nutriflex Lipid Special 1875 mL (Nutriflex LS). The following parameters: pH, osmolality, zeta potential, and particle size were determined upon sample preparation and after four hours of storage in order to determine the possible interactions. The addition of CFL to PN admixtures showed no color change, no signs of lipid emulsion destabilization, no precipitation, and no pH changes. CFL caused concentration-dependent changes in the osmolality, zeta potential, and particle size of the lipid emulsion. Only in the case of CFL-Nutriflex LS samples, the appearance of the second fraction of lipid particles was not observed. For the remaining tested samples, the second fraction of particles appeared immediately after adding CFL to the PN admixture (Kabiven and Olimel) or after 4 h of storage (Nutriflex OS and Smofkabiven). The obtained results are presented in Table 2.

## 3. Discussion

Safe pharmacotherapy depends on the administration of compatible and stable drugs. For years it has been postulated that PN admixture can be a drug carrier. This type of assumption is based on the need to conduct drug stability studies in PN admixture and to determine their interactions.

Literature data show differences in compatibility and stability results regarding cephalosporins co-administered with another drug. Research performed by Servais et al. [49] demonstrated that CFZ showed physical incompatibilities with vancomycin, nicardipine, midazolam, and propofol. The chemical incompatibility with N−acetylcysteine was observed as a linear decrease in CFZ content during storage at the temperature of 25 °C [49]. On the contrary, CFZ was compatible with several drugs, including aminoglycosides, fluconazole, ketamine, sufentanil, valproic acid, furosemide, and urapidil. Elmore et al. [50] evaluated compatibility studies between ciprofloxacin and 22 different drugs. CFX and CFZ were incompatible with ciprofloxacin solution due to the appearance of sediment and pH change of more than one unit for CFX and CFZ, respectively. Linezolid was evaluated to determine its compatibility with three cephalosporins: CFZ, CFL, and CFX [51]. All cephalosporins were physically compatible with linezolid, and only two first chemically stable during storage at the temperature of 25 °C. In contrast, CFX mixed with metronidazole was stable for 3 days at the temperature of 25 °C [52]. Another analysis proved the incompatibility of CFX mixed with labetalol [53], and CFE with piritramid [54]. Literature data regarding the compatibility of cephalosporins and PN admixtures are limited. Bouchoud et al. [34] evaluated the physical compatibility between 25 intravenous medications co-administered with Lipoflex Special. Despite observed discoloration CFX was assumed to be compatible with Lipoflex Special. Whereas Staven et al. [45] evaluated compatibility of drugs with two pediatric PN admixtures i.e., Olimel N5E and Numeta G16E. CFZ showed no signs of precipitation immediately after mixing with PN admixture or after 4 h of storage. However, after 24 h a strong color change and precipitation were observed. It was suggested that the decomposition was accelerated by the PN admixture’s components.

Our stability studies of CFE, CFU, CFX, and CFZ with PN admixture indicated a different influence of PN components on the stability of the drugs. Cephalosporins belong to the group of time-dependent antimicrobial agents. The most suitable pharmacokinetic/pharmacodynamic (PK/PD) parameter defining their activity is time with serum concentration higher than the minimum inhibitory concentration (T > MIC). For this reason, continuous infusion is preferable for this group of medications. On the other hand, the infusion of PN admixtures lasts from 16 to 24 h, depending on the indications. Due to the long duration of the PN admixture administration, it was assumed that the tested samples should meet the acceptance criteria for at least 24 h. The chemical stability studies of cephalosporins in PN admixtures (stored in one container) were carried out longer (5 days for CFE and CFU) and (7 days for CFX and CFZ) to determine the ability to store such pharmaceutical preparation (PN admixture containing antibiotics) prior to patient administration. The assumed acceptance criterion was to maintain the cephalosporin content above 90%. This criterion was met by CFX and CFZ within 24 h (under all studied conditions), while in the case of CFE and CFU, the decrease in the drug content > 10% was observed within the first 24 h. At the same time, it should be emphasized that neither CFX nor CFZ caused any signs of destabilization of the lipid emulsion or exceeding the physicochemical parameters of PN admixtures beyond the established criteria, both 24 and 96 h after sample preparation.

For antibiotics that proved to be unstable in PN admixture (CFE and CFU), additional stability studies after reconstitution in the most commonly used infusion fluids for 5 days period were performed. To expand available stability data, we decided to use, apart from water for injection, 0.9% sodium chloride, 5% glucose, additionally 10% glucose, and Ringer’s solution. Both antibiotics showed greater stability at the temperature of 5 ± 2 °C with light protection and were more susceptible to degradation during storage at the temperature of 25 °C with light exposure.

The obtained results concerning the stability of the tested cephalosporins extend the literature data [22,23,38,55,56,57,58,59], which are presented in Table 3. So far, the Stability of CFZ and CFX has been determined in reconstitution solutions. Stiles et al. [26] analyzed CFZ solutions dissolved in water for injection in a glass vial and PCV reservoirs and concluded that CFZ was stable for 7 days at the temperature of 3 °C. Stability decreased during storage at the higher temperature. The drug was stable only for 24 h if kept at a temperature of 25 °C [38,49], which is sufficient time to administer it to the patient. CFX showed longer stability in reconstitution diluents than CFZ. Solutions of CFX in 5% dextrose and normal saline i were stable for 2 days at room temperature and for 14 days at the temperature of 4 °C when CFZ solutions in either diluent were stable for only 1 day when stored at room temperature and for at least 4 days at the temperature of 4 °C [60].

Lack of compatibility data eliminates the possibility of co-administering drugs using a Y-site connector. This type of practice is often used in a situation of a limited number of intravenous accesses. The use of drug co-administration may occur, e.g., in parenterally fed patients who are administered PN admixture with Broviac or Hickman single-lumen, tunneled central catheters, and who concurrently have contraindications for other central or peripheral access. Restrictions in placing subsequent intravenous accesses result from an infection of the catheter or cannula insertion site, anatomical changes in the puncture site, thrombosis within the planned vein, and coagulopathy. In the case of peripheral vessels, the most common contraindications for intravenous cannula insertion are vascular fragility of tissue fibrosis resulting from multiple punctures, as well as dehydration and shock [61].

The mentioned clinical situations limit the possibility of administering drugs using separate infusion lines and force their co-administration. Such delivery is possible using a Y-site connector allowing co-infusion of two drugs simultaneously or by adding one drug to another and delivering them from one package. Both solutions come with the risk of drug interactions. In the first case, they may occur in the infusion line. The contact time of both drugs is short and depends on the infusion rate and the length of the common part of the infusion set. The research methodology for this type of drug interaction is based on the study of physicochemical changes at predetermined time points. The literature data show that the most frequently used time points are immediately after the samples’ preparation and after 4 h. The second time point does not derive from the real contact time of the two drugs in the infusion line but allows investigating interactions that are time-dependent and increase over time. In the compatibility studies of drugs administered via a Y-site connector, it is not necessary to assess the changes in the content of both drugs, as the short contact time should not affect their chemical stability. At the same time, it is necessary to adopt appropriate acceptance criteria for the applied research methods, which will adequately determine the safety of simultaneous infusion of the co-administrated drugs.

The compatibility of CFL in the concentration usually used to treat upper respiratory tract infections was determined with five commercially produced PN admixtures. The investigation of PN admixtures available in most countries in the world allows the wide use of obtained results. Analysis of the acceptance criteria allows to state that CFL was compatible only with Nutriflex LS, and when combined with the other PN admixtures, it caused the formation of lipid particles above 500 nm. The presence of lipid droplets exceeding the pharmacopeial limits (MDD < 500 nm) [2] disqualifies the possibility of the administration of CFL simultaneously with Kabiven, Smofkabiven, Olimel, and Nutriflex OS via a Y-site connector. No data on the compatibility of CFL with PN admixture was found in the available literature. However, information about the Stability of CFL in infusion fluids (water for injection, 0.9% sodium chloride, 5% glucose), stored at different temperatures (−20 to 37 °C), at different times and in different packages (elastomeric pump, syringe, PVC bag) are available. Compatibility of CFL with piritramide [54] or linezolid was also determined [51], and detailed data on CFL stability and compatibility are given in Table 3 [21,25,26,51,54,55,60,62,63].

**Table 3:** Stability and compatibility literature data of selected cephalosporins.

Drug	Stability and Compatibility Results	Brand Name	References
Cefuroxime sodium (CFU)	CFU was stable for 7 days at 3 °C in water for injection.	Zinacef, Glaxo Inc, Philadelphia, PA, USA	[22]
	A drug stored in 0.9% sodium chloride over 4 months at −18 °C was stable.	Cefuroxime Sandoz, Basel, Switzerland	[23]
	The drug was stable for 13 days at 4 °C stored in polyvinyl chloride (PVC) bags.	Zinacef, Glaxo Inc, USA	[55]
	During simulated Y-site administration, the drug was compatible with propofol injectable emulsion for one hour at 23 °C.	No data, Lilly, Indiana, IN, USA	[62]
	CFU was incompatible with ciprofloxacin.	Zinacef, Glaxo Wellcome Inc, Middlesex, UK	[50]
Ceftazidime pentahydrate (CFZ)	CFZ was stable for 10 days at 3 °C in water for injection.	Ceptaz, Glaxo Inc, Philadelphia, PA, USA	[22]
	CFZ was stable for 24 h at a temperature of 25 °C;	No data	[49]
	CFZ in glucose and normal saline was stable for 1 day, stored at room temperature, and for 4 days at 4 °C.	No data, GlaxoSmithKline, Mississauga, Canada	[60]
	CFZ in sterile water in either glass vials or plastic syringes was stable for 8 h at room temperature or 96 h at 4 °C.	Fortaz, Glaxo Inc, USA	[24]
	CFZ is sterile water for injection was stable at −20 °C for 30 days, thawed at 5 °C for 4 days, and at 37 °C for a day.	No data, Glaxo Inc, USA	[26]
	CFZ in sterile water in either glass vials or plastic syringes is stable for 8 h at room temperature or 96 h at 4 °C.	Fortaz, Glaxo Inc, USA	[24]
	CFZ was stable in water for injection stored over 24 h at 25 °C.	Glazidim; Glaxosmithkline, Rixensart, Belgium	[38]
	During simulated Y-site administration, the drug was compatible with propofol injectable emulsion for one hour at 23 °C.	No data, SmithKline Beecham, Philadelphia, PA, USA	[62]
	CFZ was compatible with amikacin, tobramycin, gentamycin, fluconazole, ketamine, sufentanil, valproic acid, morphine, urapidil, furosemide, adrenaline, insulin, methylprednisolone; CFZ was incompatible with N−acetylcysteine, dobutamine, nicardipine, theophylline, piritramide, phenytoin, midazolam, propofol, clarithromycin, erythromycin, and vancomycin.	Glazidim; GlaxoSmithkline, Rixensart, Belgium	[63]
	CFZ and linezolid were physically compatible and chemically stable for at least 7 days, stored at 4 °C, and for 3 days at 23 °C protected from light.	No data, McNeil Pharmaceutical, Ohio, OH, USA	[51]
	The drug was compatible with Olimel N5E and Numeta G16E.	No data, Fresenius Kabi, Uppsala, Sweden	[45]
	CFZ was incompatible with ciprofloxacin.	Tazicef, SmithKline Beecham, USA	[50]
Cefepime dichlorohydrogen monohydrate (CFE)	CFE was stable in normal saline over 24 h at room temperature.	No data	[25]
	CFE was stable in water for injection stored over 20.5 h at 25 °C and 13 h at 37 °C.	Maxipime, Bristol-Myers-Squibb, Brussels, Belgium	[38]
	Cefepime was most stable in the pH range 4 to 6.	Bulk material, Bristol-Myers-Squibb, New York, NY, USA	[56]
	C diluted with 0.9% sodium chloride or 5% glucose in polyethylene containers showed stability for 48 h at 24 in daylight or 15 days at 4 ± 2 °C in the dark.	Axepim Bristol-Myers-Squibb, Rueil-Malmaison, France	[57]
	CFE was stable up to 2 days in the solutions stored at 22–24 °C.	Maxipime, Bristol-Myers-Squibb, USA	[58]
	The drug was stable in normal saline for 2 days at 23 °C.	No data, Bristol-Myers-Squibb, USA	[59]
	CFE was compatible with amikacin, tobramycin, gentamycin, vancomycin, fluconazole, ketamine, sufentanil, valproic acid, morphine, urapidil, furosemide, insulin, methylprednisolone; CFE was incompatible with N−acetylcysteine, dobutamine, nicardipine, theophylline, piritramide, phenytoin, midazolam, propofol, and vancomycin.	Maxipime, Bristol-Myers-Squibb, Belgium	[63]
	The drug was compatible with Nutriflex Lipid Special parenteral nutrition admixture.	Cefepime Orpha, Orpha Pharma, Küsnacht, Swizetland	[34]
Ceftriaxone sodium (CFX)	Drug solutions in glucose and normal saline were stable for 2 days at room temperature and 14 days at 4 °C.	No data, Mississauga, Roche, Canada	[60]
	CFX was accelerated decomposed after mixing with linezolid.	No data, Ortho-McNeil Pharmaceutical, Raritan, NJ, USA	[51]
	The drug was incompatible with labetalol.	No data, Roche, Indiana, IN, USA	[53]
	During simulated Y-site administration, the drug was compatible with propofol injectable emulsion for one hour at 23 °C.	No data, Roche, USA	[62]
	CFX mixed with metronidazole was stable for 3 days at 25 ± 1 °C.	No data	[52]
	Ceftriaxone and metronidazole mixed at concentrations of 20 and 15 mg/mL, respectively, immediately formed precipitates.	Rocephin, Roche, USA	[64]
	CFL was stable in normal saline over 24 h at room temperature.	No data	[25]
Cefazolin sodium (CFL)	CFL in sterile water for injection was stable at −20 °C for 30 days, thawed at 5 °C for 4 days, and at 37 °C for one day.	No data, Smith Kline and French Laboratories, Philadelphia, PA, USA	[26]
	CFL in glucose or normal saline was stable at least 3 days at room temperature and for at least 26 days at 4 °C.	No data, Novopharm, Markham, ON, Canada	[60]
	The drug was stable in normal saline stored at 5 °C for 22 days and at 25 °C for 7 days.	No data	[21]
	CFL stored in polypropylene syringes or PVC minibags was stable for up to 30 days stored at 5 °C with light protection, followed by an additional 72 h at 21 °C to 25 °C with exposure to light.	No data	[65]
	CFL was stable for at least 30 days at 4 °C.	Cefacidal, Bristol-Myers, Roma, Italy	[55]
	During simulated Y-site administration, the drug was compatible with propofol injectable emulsion for one hour at 23 °C.	Marsam, New Britain, CT, USA	[62]
	CFL precipitated with piritramid solutions.	No data	[54]
	CFL with linezolid was physically compatible and chemically stable for at least 7 days, stored at 4 °C protected from light.	No data, Bayer Corporation, Whippany, NJ, USA	[51]

## 4. Materials and Methods

### 4.1. Materials

Drugs used in this research were: cefuroxime sodium (Zinacef 1500 mg, GlaxoSmithKline Ltd., London, UK), cefepime dichlorohydrogen monohydrate (Cefepime Kabi 2 g, Fresenius Kabi AB, Uppsala, Sweden), ceftriaxone sodium salt (Tartriakson 1 g, Polfa Tarchomin, Warszawa, Poland), ceftazidime pentahydrate (Biotum 2 g from Polpharma S.A., Starogard Gdański, Poland), and cefazolin sodium salt (Tarfazolin 1 g, Tarchomińskie Zakłady Farmaceutyczne Polfa, Warsaw, Polska). All cephalosporins were powder for solution for injection. Chloroform (POCh, Gliwice, Poland) was used for breaking up the lipid emulsion. Other chemicals were used in HPLC analysis and were obtained from Merck, Darmstadt, Germany: ammonium dihydrogen phosphate, acetonitrile for HPLC, acetic acid, potassium dihydrogen phosphate.

The following pharmaceutical preparation was used to prepare the PN admixture: Aminoplasmal B. Braun 10% E, 40% Glucose B. Braun, Lipofundin MCT/LCT 20, Water for injection (all purchased from B. Braun Melsungen AG, Melsungen, Germany), Kalium Chloratum 15% WZF (WZF Polfa S.A., Warsaw, Poland), Natrium Chloratum 10%, Inj. Magnesii sulfurici 20% (purchased from Polpharma S.A., Starogard Gdański, Poland), Calcium gluconate 10% (Added Pharma, Oss, The Netherlands) and Glycophos (Fresenius Kabi AB, Uppsala, Sweden).

The following parenteral infusion fluids were purchased from B. Braun Melsungen AG (Melsungen, Germany): 5% Glucose, 10% Glucose, 0.9% Natrium chloratum, Ringer solution.

Five ready-to-use PN admixtures were used in this study: Kabiven 1540 mL (Kabiven), Fresenius Kabi AB, Uppsala, Sweden; Smofkabiven 1477 mL (Smofkabiven), Fresenius Kabi AB, Sweden; Olimel N9E 1500 mL (Olimel) Baxter, Poland; Nutriflex Omega Special 1875 mL (Nutriflex OS) B. Braun Melsungen AG, Melsungen, Germany; and Nutriflex Lipid Special 1875 mL (Nutriflex LS) B. Braun Melsungen AG, Germany.

### 4.2. Analysis of CFU and CFE

A total of 1500 mg of Zinacef in 12 mL and 2000 mg of Cefepime Kabi were dissolved in 10 mL of water for injection. A total of 3 mL of each solution were taken and transferred to infusion fluids and PN admixture. The composition of the PN admixture is presented in Table 4. Every 24 h, 0.5 mL samples of cephalosporin solutions in the infusion fluids were withdrawn and diluted with water for injection to a volume of 2 mL.

To 4 mL sample withdrawn from the PN admixture, 2 mL of chloroform was added, shaken 10 min, and then centrifuged 15 min (5600 rpm). The supernatant was collected from centrifuged samples, filtered through a membrane filter, and then 0.15 mL was collected, to which 1.5 mL of water for injection was added. The samples prepared in this way were injected onto the chromatographic column, and the HPLC analysis was performed. The separation parameters were as follows: column LiChrospher 100 RP-18, end-capped, 5 µm; mobile phase: acetate buffer-methanol 75:25 (V/V); flow rate: 1.2 mL/min for CFU and 0.6 mL/min for CFE; column temperature: 25 °C; detector wavelength: 272 nm; injection volume: 10 µL.

### 4.3. Analysis of CFX and CFZ

Tartriaxone (CFX) and Biotum (CFZ) were reconstituted according to the summary of product characteristics. Drugs were diluted with water for injection and added to PN admixture to the concentration of 0.8 mg/mL and 2.4 mg/mL for CFX and CFZ, respectively. The composition of the PN admixture is presented in Table 4. Upon drug addition to PN admixtures (t = 0 h) and after every 24 h, the 3.0 mL samples were withdrawn and introduced into a plastic vial, and 1.0 mL of chloroform was added. After agitation for 15 min (MPW Med. Instruments, Warsaw, Poland) and centrifugation for 30 min at a rate of 5800 rpm (GFL 3005, Lab Unlimited U.K., Camberley, U.K.), the water layers were filtered through a 0.2 µm membrane filter. Each sample was prepared in triplicate and examined in order to determine the influence of temperature and light exposure during storage on the properties of PN admixtures and drug contents. The PN admixture was stored in three different conditions: at the temperature of 5 ± 1 °C with light protection, at the temperature of 25 ± 1 °C with light protection, and the temperature of 25 ± 1 °C with light exposure. Long-term stability studies at low temperature at 4 ± 1 °C without light access (simulating storage in the refrigerator) or 25 ± 2 °C with and without light access (simulating storage in the room temperature) allows preparing drug solution in hospital pharmacy and storing them before use. This practice spare costs and enhance safety because of preparation under aseptic condition. Light-sensitive drugs require special packaging with a UV filter. As a result, we were obliged to check stability during administration with and without access to light.

The HPLC analytical apparatus comprised a 1220 Infinity LC chromatography system equipped with a DAD detector, a G1315C optical unit, a binary pump, an autosampler, and a column oven (Agilent Technologies, Santa Clara, CA, USA). The separation condition is detailed in Table 5.

### 4.4. Analysis of CFL

The compatibility of five different ready-to-use PN admixtures (Kabiven, Smofkabiven, Olimel, Nutriflex LS, and Nutriflex OS) and CFL solution in three different volume ratios of 1:1, 2:1, and 4:1 was tested. The procedure of sample preparation was similar to our previous investigations concerned with the compatibility studies of PN admixtures and meropenem, vancomycin, or colistin [11,33,61]. Briefly, PN admixtures were activated and supplemented in aseptic conditions. The CFL drug product (Tarfazolin 1 g, Tarchomińskie Zakłady Farmaceutyczne Polfa, Warsaw, Polska) was reconstituted by dilution with 10 mL of water for injection, and the obtained solution was transferred from the vial to 100 mL ecoflac bottle with normal saline (Natrium Chloratum 0.9%, B. Braun Melsungen AG, Melsungen, Germany). The concentration of CFL in prepared infusion was 9.09 mg/mL.

PN admixtures and CFL solutions were mixed in ratios 1:1, 2:1, and 4:1. The drug-PN admixture ratios were calculated based on their infusion rates, reproducing daily clinical practice.

Samples were prepared by mixing the appropriate volume of supplemented PN admixtures and CFL solution in a 10 mL plastic vial. Each sample was prepared in triplicate and examined immediately after preparation and after 4 h of storage at the temperature of 25 ± 1 °C. The same parameters were measured for supplemented PN admixtures with vitamins and trace elements, as well as for samples obtained by mixing supplemented PN admixtures with CFL in studied ratios.

### 4.5. Characteristic of PN Admixture

Characteristics of the physicochemical properties of PN admixtures without drug and containing the tested drugs were performed. The procedure was the same for PN admixture containing CFX, CFZ, and CFL. Following the European Pharmacopoeia [41], PN admixtures were visually assessed for the presence of visible particles or color change. Visual inspection was performed against a black-and-white contrast background by two observers. Following the pharmacopoeial requirements for intravenous lipid emulsions, to consider PN admixtures as compatible with studied cephalosporines, the following criteria must be met: practically free from visible particles; no precipitation can be detected by any of the observers upon visual inspection.

The pH was measured at room temperature using a Mettler Toledo Seven Compact pH/ion S220 pH-meter, and the osmolality was measured at room temperature using an 800 CL TridentMed osmometer. In accordance with Bouchoud et al. [10], we set up the acceptance criterion of ΔpH ≤ 0.2 for the pH change during the study period. The acceptance limit for osmolality changes was set as <5%. The changes in the pH and osmolality exceeding this value may evidence the acid-base changes in the solution (hydrolysis of ingredients) or precipitation.

The particle size of lipid emulsion and zeta potential (ξ) of PN admixtures were measured at the temperature of 25 °C using a Zetasizer Nano ZS (Malvern Instruments, Malvern, U.K.) by dynamic light scattering (DLS) and laser Doppler velocimetry, respectively. The sample preparation, particle size, and zeta potential determination were performed according to the methodology described in our previous work [42,43,44]. The results of droplet diameter measurements were presented as MDD (intensity-weighted mean droplet diameter), dF1 (the diameter of the particles present in the highest intensity in the first fraction), and dF2 (the diameter of the particles present in the highest intensity in the second fraction). The homogeneity of the samples was determined by the polydispersity index (PDI). All the measurements were performed in triplicate, and the results were expressed as average ± standard deviation. To consider the PN admixtures to be compatible with studied cephalosporins, the size of lipid droplets expressed as intensity-weighted MDD cannot exceed the pharmacopeial limit of 500 nm. This criterion was set for the U.S. Pharmacopeia Method I for the determination of the mean droplet size of lipid injectable emulsions [2]. The measuring apparatus was calibrated before use according to the manufacturer’s instructions. As a positive control, we used PN admixtures without the addition of vitamins and trace elements upon preparation. The negative control was PN admixtures subjected to stress factors (exposure to 150 °C for 30 min or addition of 0.1 mol/L HCl at 1:1 volume ratio).

### 4.6. Statistical Analysis

ANOVAs were used to determine the statistical significance between samples. The a priori level of significance was *p* < 0.05.

## 5. Conclusions

Our studies allowed us to assess the stability and compatibility of five cephalosporins in different research models.

CFE and CFU were added to the infusion fluids and the PN admixture decomposed during storage. This process was intensified at the temperature of 25 ± 2 °C and exposure to light. Both antibiotics were stable in all infusion fluids tested (water for injection, 0.9% sodium chloride, 5% and 10% glucose, and Ringer solution) for a minimum of 48 h. The content of CFE and CFU added to PN admixture after 24 h decreases below 90%, which disqualifies the possibility of adding these drugs to such a medium. The content of CFX and CFZ in PN admixture after 24 h was >90%, which makes it possible to recommend this method of administration for these drugs.

CFL administered simultaneously with PN admixture by the same infusion set using a Y-site was compatible only with Nutriflex LS. For the remaining examined, PN admixtures (Kabiven, Olimel, Nutriflex OS, and Smofkabiven) caused an increase in the MDD above 500 nm, which disqualifies the possibility of simultaneous infusion.

## Figures and Tables

**Table 1:** Stability of CFE and CFU in parenteral infusion fluids and PN admixtures.

Time, h	Water for Injection	0.9% Sodium Chloride	5% Glucose	10% Glucose	Ringer’s Solution	PN Admixtures
	Drug content (%)					
	CFE stored at 5 ± 1 °C with light protection					
0	100.00	100.00	100.00	100.00	100.00	100.00
24	96.56	98.73	97.38	97.28	99.43	86.10
48	93.51	98.00	95.53	92.88	96.90	83.41
72	90.23	96.90	95.26	90.11	91.06	79.62
96	88.38	88.45	92.69	87.92	90.24	77.18
	**CFE stored 25** **±** **2 °C with light protection**					
0	100.00	100.00	100.00	100.00	100.00	100.00
24	94.83	94.94	91.38	99.00	93.05	73.87
48	94.27	91.09	89.50	90.30	90.72	65.91
72	85.79	89.27	84.72	85.56	88.44	59.67
96	82.86	86.72	82.41	86.30	83.59	54.21
	**CFU stored at 5** **±** **1 °C with light protection**					
0	100.00	100.00	100.00	100.00	100.00	100.00
24	98.99	99.55	99.75	92.64	97.93	74.57
48	94.45	91.63	97.97	91.05	95.59	59.87
72	86.32	79.13	94.8	76.36	81.17	42.97
96	77.31	63.64	77.17	70.44	76.36	38.27
	**CFU stored 25 ± 2 °C with light protection**					
0	100.00	100.00	100.00	100.00	100.00	100.00
24	99.58	90.32	98.93	97.81	91.63	73.71
48	94.88	83.53	98.75	53.92	77.00	54.06
72	90.63	77.85	69.07	44.84	48.37	43.36
96	69.32	64.27	49.66	32.60	46.04	24.65

**Table 2:** Characteristic of CFL-PN admixtures samples.

Sample	Ratio	pH *		Osmolality ± SD		Zeta Potential ± SD		PDI		dF_1_		dF_2_		MDD	
				(mOsm/kg H_2_O)		(mV)				(nm)		(nm)		(nm)	
		0 h	4 h	0 h	4 h	0 h	0 h	0 h	0 h	0 h	4 h	0 h	4 h	0 h	4 h
CFL-Kabiven	1:1	5.48	5.49	683 ± 1	683 ± 6	−12.1 ± 0.3	−13.9 ± 0.2	0.15 ± 0.01	0.14 ± 0.02	326 ± 3	328 ± 4	–	–	281 ± 6	281 ± 6
	2:1	5.49	5.5	537 ± 4	538 ± 4	−14.2 ± 0.4	−12.9 ± 0.4	0.16 ± 0.02	0.16 ± 0.01	320 ± 3	320 ± 10	–	–	278 ± 6	278 ± 6
	4:1	5.48	5.5	441 ± 1	438 ± 2	−13.5 ± 0.7	−14.8 ± 0.5	0.16 ± 0.01	0.16 ± 0.01	325 ± 3	331 ± 6	1243 ± 8	1443 ± 7	278 ±11	289 ± 11
CFL-Nutriflex LS	1:1	5.47	5.45	984 ± 2	995 ± 3	−12.2 ± 0.1	−23 ± 1.4	0.06 ± 0.02	0.07 ± 0.02	233 ± 3	228 ± 1	–	–	219 ± 2	211 ± 2
	2:1	5.47	5.46	735 ± 1	739 ± 1	−14 ± 0.2	−12.6 ± 0.4	0.06 ± 0.04	0.06 ± 0.02	229 ± 4	229 ± 6	–	–	213 ± 5	213 ± 2
	4:1	5.47	5.47	547 ± 4	549 ± 1	−19.7 ± 1	−27.7 ± 1.2	0.07 ± 0.03	0.08 ± 0.03	232 ± 7	226 ± 0	–	–	215 ± 3	208 ± 2
CFL-Olimel	1:1	6.27	6.26	820 ± 4	865 ± 5	−10.8 ± 0.4	−12.9 ± 0.7	0.11 ± 0.01	0.12 ± 0.04	287 ± 5	280 ± 6	–	1662 ± 2	257 ± 2	256 ± 5
	2:1	6.27	6.26	612 ± 1	639 ± 3	−15 ± 0.3	−11.8 ± 0.7	0.13 ± 0.01	0.12 ± 0.01	296 ± 8	293 ± 4	–	–	259 ± 4	259 ± 4
	4:1	6.24	6.29	476 ± 2	511 ± 1	−10.9 ± 0.5	−13.7 ± 1.5	0.13 ± 0.02	0.24 ± 0.01	287 ± 5	284 ± 4	1728 ± 3	3537 ± 8	260 ± 6	280 ± 0
CFL-Nutriflex OS	1:1	5.62	5.59	971 ± 4	1008 ± 1	−9.3 ± 0.1	−9.9 ± 0.1	0.09 ± 0.01	0.57 ± 0.04	243 ± 4	343 ± 4	–	5005 ± 7	222 ± 3	550 ±11
	2:1	5.59	5.59	728 ± 2	846 ± 1	−11.3 ± 0.4	−11.9 ± 0.1	0.07 ± 0.01	0.57 ± 0.09	244 ± 3	1469 ± 4	–	1970 ± 6	225 ± 1	663 ± 4
	4:1	5.6	5.59	531 ± 1	557 ± 6	−13.1 ± 1	−15.7 ± 0.9	0.10 ± 0.02	0.10 ± 0.02	237 ± 5	240 ± 6	–	–	217 ± 1	218 ± 1
CFL-Smofkabiven	1:1	5.45	5.46	916 ± 4	891 ± 5	−9.4 ± 0.7	−9.7 ± 0.3	0.09 ± 0.02	0.13 ±0.001	277 ± 1	271 ± 9	–	–	249 ± 4	237 ± 2
	2:1	5.45	5.47	686 ± 1	668 ± 3	−9.8 ± 0.4	−9.6 ± 0.3	0.12 ± 0.01	0.12 ± 0.02	276 ± 7	270 ± 7	–	–	247 ± 4	240 ± 3
	4:1	5.46	5.48	521 ± 4	505 ± 2	−12.6 ± 0.4	−11.6 ± 0.1	0.11 ± 0.01	0.15 ± 0.02	264 ± 5	250 ± 7	–	3454 ± 8	236 ± 1	231 ± 1

* SD of pH values of all samples were below 0.01. SD—standard deviation, PDI—polydispersity index, dF1—diameter of the particles present at the highest intensity in the first fraction, dF2—diameter of particles present at the highest intensity in the second fraction, MDD—intensity-weighted mean droplet diameter, CFL—cefazoline, PN—parenteral nutrition.

**Table 4:** Composition of studied PN admixture without drug.

Ingredient	Pharmaceutical Preparation	Unit	PN A	PN B
Amino acids	Aminoplasmal BB 10% E	g/L	20	24
Lipid emulsion	Lipofundin MCT/LCT 20%		70	88
Carbohydrates	Glucose 40%		24	24
Sodium	Natrium chloratum 10%	mmol/L	45	41
Potassium	Kalium chloratum 15%		20	16
Calcium	Calcium gluconate 10%		2.5	1.8
Phosphates	Glycophos		15	8
Magnesium	Inj. Magnesii sulfurici 20%		1.5	2.5

PN A was used for CFE and CFU; PN B was used for CFX and CFZ.

**Table 5:** Chromatographic separation conditions of CFX and CFZ.

Factor	CFZ	CFX		
Column	C18 with a precolumn C18; LiChrospher 100, (5 µm); 4 mm i.d., 150 × 4.6 mm; Merck Darmstadt Germany	C18 with a precolumn C18; LiChrospher 100, (5 µm); 4 mm i.d.; 250 × 4.6 mm; Merck Darmstadt Germany		
Column temperature	25 °C	40 °C		
Mobile phase	Phase A: 0.5 mL 12% acetic acid, 50 mL 0.2 mol/L potassium dihydrogen phosphate buffer, 50 mL acetonitrile, and up to 1000 mL water Phase B: 0.5 mL 12% acetic acid, 50 mL 0.2 mol/L potassium dihydrogen phosphate buffer, 400 mL acetonitrile and up to 1000 mL water	Phase A: Acetonitrile Phase B: 50 mmol/L dihydrogen ammonium phosphate solution		
Elution	Isocratic 95:5 ratio A:B	Gradient		
		Time, min	Mobile phase A (%, V/V)	Mobile phase B (%, V/V)
		0–20	100 → 65	0 → 35
		21–25	65 → 100	35 → 0
Flow rate	1 mL/min	1 mL/min		
Detection	255 nm	254 nm		
Injection	10 µL	10 µL		
Run time	25 min	25 min		

## Data Availability

Data are contained within the article.
